# Effect of non-surgical periodontal treatment on serum albumin levels in patients with chronic periodontitis

**DOI:** 10.15171/japid.2018.004

**Published:** 2018-06-20

**Authors:** Adileh Shirmohammadi, Masoumeh Faramarzi, Ashkan Salari, Mehrnoosh Sadighi Shamami, Amir Reza Babaloo, Zohreh Mousavi

**Affiliations:** ^1^Department of Periodontics, Dental School, Tabriz University of Medical Sciences, Tabriz, Iran; ^2^Department of Periodontics, Dental School, Rasht University of Medical Sciences, Rasht, Iran; ^3^Private Practice, Tabriz, Iran

**Keywords:** Albumin, chronic periodontitis, dental scaling, non-surgical inflammation, root planing

## Abstract

**Background:**

Albumin is a protein whose serum levels decrease in inflammatory conditions such as periodontal diseases. This study was undertaken to evaluate changes in serum albumin levels in patients with and without periodontal diseases prior and subsequent to non-surgical periodontal treatment and its relationship with clinical parameters of periodontal disease.

**Methods:**

Twenty patients diagnosed as having chronic periodontitis and 20 periodontally healthy subjects, referring to Tabriz Faculty of Dentistry, were selected. Serum albumin levels and clinical variables of periodontal disease (probing pocket depth, gingival index, bleeding index, clinical attachment level and plaque index) were determined before treatment and three months subsequent to non-surgical periodontal treatment. Data were subjected to descriptive statistical analyses (mean ± SD). Serum levels of albumin and clinical parameters were compared between the two groups with independent-samples t-test. Paired-samples t-test was applied to compare the variables before and after treatment in the case group. Statistical significance was defined at P<0.05.

**Results:**

The mean serum albumin level of chronic periodontitis patients (3.62±0.11 mg/dL) exhibited a significantly lower value compared to subjects who were periodontally healthy (4.17±0.29 mg/dL), with the serum albumin levels increasing significantly three months postoperatively (3.78±0.33 mg/dL), approaching the level in subjects who were periodontally healthy (P<0.05).

**Conclusion:**

Decreases and increases in serum albumin levels under the effect of periodontal disease and its treatment indicated an inverse relationship between the albumin levels of serum and chronic periodontitis.

## Introduction

Periodontal diseases are inflammatory conditions of tooth-supporting structures, induced by subgingival anaerobic gram-negative bacteria. This progressive condition is characterized by periodontal tissue destruction.^1^ Periodontitis is highly prevalent all over the world and is an important health challenge in different countries.^2,3^ Early diagnosis and treatment play a key role in the prevention of periodontitis progression.^4^

Currently, the diagnosis of periodontitis almost completely relies on a number of clinical parameters such as pocket depth (PD), bleeding on probing (BOP), clinical attachment level (CAL) and radiographic findings.^5^ These measurements for the diagnosis of periodontitis usually have limited efficacy because they predominantly indicate periodontal disease activity in the past, not the ongoing disease activity. It is necessary to develop new diagnostic tests in order to determine the presence of active disease, predict its progression in future and evaluate response to periodontal treatment by assessing improvements in clinical parameters of the periodontium. Advances in research on the diagnosis of periodontal disease exhibit trends toward techniques that can identify periodontal risks with the use of objective measurements such as biomarkers. Biomarkers are synthesized and secreted by both healthy and systemically affected subjects. Biomarkers have the potential for use to assess the health, initiation of the disease, the response to treatment and treatment outcomes. Serum albumin is one of these biomarkers.^6^

Albumin is a protein, the concentration of which undergoes changes in inflammatory conditions such as hepatic and renal conditions.^7^ In chronic diseases in which inflammatory cytokines such as IL-1, IL-6 and α-factor are released, serum albumin levels decrease.^8^ A large number of researchers has reported a relationship between a decrease in serum albumin levels and patient mortality.^9,10^ Mojon et al^11^ reported that individuals with a mean age of 85 years, who have pockets measuring >6 mm in depth exhibit significantly lower serum levels of albumin In addition, studies have shown that there is an association between severe periodontitis and changes in the serum levels of inflammatory markers.^12-14^ However, some other studies have yielded contradictory Results.^15,16^ Due to the unavailability of studies on the relationship between serum albumin levels and periodontitis, this study was designed to evaluate changes in serum albumin levels in subjects with periodontal disease and healthy subjects prior and subsequent to non-surgical periodontal treatment and the relationship between the relevant clinical variables and periodontal disease

## Methods

### Sample size and sampling 

Based on a pilot study before this study, the serum albumin levels were determined in 5 subjects as a control group and in 5 patients with chronic periodontitis at before and 3 months after non-surgical periodontal treatment. According to the results and by considering α=0.05 and a statistical power of 80%, the sample size was estimated at 16 subjects in each group.

### The target population 

The subjects were selected from those referred to the Department of Periodontics, Tabriz Faculty of Dentistry. The subjects consisted of 40 individuals (20 male and 20 female subjects) with an age range of 25‒56 years. All the subjects were systemically healthy, did not smoke and had a minimum of 20 teeth in their oral cavities.

### Procedural steps

The present clinical trial was carried out on patients referring to the Department of Periodontics, Tabriz Faculty of Dentistry. The subjects participated in this study voluntarily after the procedural steps were completely explained to them and after they signed informed consent forms. The subjects were assigned to the case (chronic periodontitis) and healthy control groups after diagnostic examinations and by considering inclusion and exclusion criteria (17).

### Inclusion criteria

The inclusion criteria for periodontally healthy subjects consisted of no loss of attachments at interdental areas, no probing depths of ≥3 mm in any tooth area, and a bleeding index of ≤10% in the whole oral cavity.^21^ The inclusion criteria in the chronic periodontitis group consisted of CAL ≥4 mm in two or more interdental areas or probing pocket depths ≥5 mm in two or more interdental areas.^22^

### Exclusion criteria

The exclusion criteria were as follows: history of taking NSAIDs and antimicrobial agent in the 6 months preceding the study, history of any periodontal treatment in the 6 months preceding the study, any systemic diseases and infectious conditions other than chronic periodontitis, aggressive periodontitis, pregnancy or breast-feeding and use of mouthwashes and vitamin supplements in the 3-month period before the study.

### Study groups

*Group 1:* This group consisted of subjects with chronic periodontitis in a minimum of 20 natural teeth, with ≥4 of CAL in two or more interdental areas or with ≥5 mm of probing pocket depths in two or more interdental areas.

*Group 2:* This group consisted of periodontally healthy subjects without CAL in interdental areas, without probing pocket depths of ≥3 mm in any area, and with ≤10% of BOP in the whole oral cavity.

Blood samples were collected from the subjects in both groups. A total of 4 mL of venous blood samples were taken at baseline (before registration of clinical parameters). These samples were transferred into vacuum sterile test tubes without anticoagulants and transferred to the laboratory in <2 hours. Serum albumin levels were determined using an enzymatic technique by an automated analyzer (Model BS-480, Mindary Co., China).

The overall periodontal status of the subjects was determined and registered by determining probing pocket depths and CALs (in 6 areas of each tooth except for third molars, consisting of mesiolingual, lingual, distolingual, distobuccal, buccal and mesiobuccal areas), bleeding index (18), gingival index and plaque index^19,20^ by one examiner using a UNC-15 hand probe (Hu-Friedy, USA). Since all the measurements were carried out by one operator, there was no inter-observer variability. To determine the reproducibility of examinations, in 10 subjects the clinical parameters were carried out twice in one session with an interval of one hour. Evaluation of the mean differences between the values yielded a kappa value of 0.81 with 90% accuracy.

Then non-surgical periodontal treatment was carried out in the group with chronic periodontitis, which consisted of oral hygiene instructions, careful SRP and use of 0.12% CHX twice a day for half a minute for two weeks (Kin Gingival Mouthwash, Cosmodent Co., Spain). SRP was rendered by an operator in one 1-hour visit. In the control group, only oral hygiene instructions were provided for the subjects. The subjects in the case groups were followed for 4, 8 and 12 weeks subsequent to periodontal treatment in order to evaluate the oral hygiene status. At the end of the 3-month follow-up period, venous blood samples were again collected from the subjects, followed by re-evaluation of clinical parameters.

### Statistical analysis

SPSS 20 was used for statistical analysis of data at P<0.05. Kolmogorov-Smirnov test was used to assess normal distribution of data; since data were distributed normally parametric tests were used to analyze differences in the means between the two study groups. Data were analyzed using descriptive statistics (mean ± SD). Independent-samples t-test was applied to compare the serum albumin levels and clinical parameters between the case and control groups. Paired-samples t-test was applied to compare these variables in the case group before and after treatment.

### Ethical considerations

All the subjects signed informed consent forms to be included in the study. The protocol of the study was approved by the Ethics Committee of Tabriz Faculty of Dentistry under the code IRCT201502077128N5.

## Results

Forty patients (20 male and 20 female patients) referred to the Department of Periodontics, Tabriz Faculty of Dentistry, were assigned to two groups based on inclusion and exclusion criteria: 20 periodontally healthy subjects (9 and 11 males and females, respectively) with an age range of 25‒42 and a mean age of 33.18±4.14 years in the control group, and 20 subjects with chronic periodontitis (11 and 9 males and females, respectively) with an age range of 28‒56 and a mean age of 42.13±8.36 years in the case group. The subjects in the case group were diagnosed as having generalized moderate-to-severe chronic periodontitis. No subject was excluded and all completed the study.

Table 1 presents the descriptive analyses of clinical and biochemical variables of the subjects at baseline. Kolmogorov-Simonov test showed normal distribution of all the data on serum albumin levels, GI, PI, GBI, CAL and PD in the control and case groups prior to and subsequent to non-surgical periodontal treatment.

**Table 1:** Clinical and biochemical parameters of the subjects at baseline

**Parameter**	**Total**	**Chronic periodontitis**	**Periodontally healthy**	**P-value**
PD (mm)	3.47±1.62	5.03±0.48	1.92±0.33	<0.001*
**CAL (mm)**	2.73±2.45	5.13±0.47	0.55±0.12	<0.001*
**GBI**	39.49±36.02	74.66±4.33	7.86±1.92	<0.001*
**GI**	1.33±0.87	2.17±0.24	0.50±0.13	<0.001*
**PI**	1.28±0.90	2.13±0.31	0.79±0.13	<0.001*
**Serum albumin (mg/dL)**	260.90±49.57	3.62±0.11	4.17±0.29	<0.001*

*P<0.05; statistically significant. Significant differences were detected in PD, CAL, GBI, GI and PI between the two groups (P<0.05). The mean serum albumin level in subjects with chronic periodontitis (3.62±0.11 mg/dL) was significantly less than that in the control group (4.17±0.29 mg/dL) (P<0.05).

At 3-month follow-up after non-surgical periodontal treatment, PD, CAL, GBI, PI and GI improved (Table 2), with significant differences in the means of all the clinical variables between the period before treatment and three months after non-surgical periodontal treatment (P<0.001).

All the patients diagnosed with chronic periodontitis exhibited significant increases in serum albumin levels three months after treatment (3.78±0.33 mg/dL) (P<0.01).

**Table 2:** Clinical periodontal parameters 3 months after non-surgical periodontal treatment

**Parameter**	**Periodontitis** **before treatment**	**Periodontitis** **after treatment**	**P-value**
**PD (mm)**	5.03±0.48	3.29±0.27	<0.001*
**CAL (mm)**	5.13±0.47	3.87±0.91	<0.001*
**GBI (%)**	74.66±4.33	17.13±4.07	<0.001*
**GI**	2.17±0.24	0.98±0.31	<0.001*
**PI**	2.13±0.31	0.81±0.30	<0.001*
**Serum albumin (mg/dL)**	3.62±0.11	3.78±0.33	<0.001*

*P<0.05; statistically significant.

## Discussion

This study was designed to evaluate the effects of periodontal disease and non-surgical periodontal treatment on serum albumin levels. The results showed lower serum albumin levels in chronic periodontitis patients compared to periodontally healthy subjects. Furthermore, a decrease in the severity of periodontal inflammation after treatment gave rise to an increase in serum albumin levels.

Periodontitis is induced by the bacterial infection of tooth-supporting structures.^23^ The host response to bacterial infection results in changes in the concentrations of acute-phase proteins and the synthesis and release of IL-1, IL-6 ad TNF-α. These proinflammatory cytokines have a key role in the destruction of periodontal tissues. The response of acute-phase proteins demonstrates the defensive and adaptive mechanisms taking place prior to immunologic responses in the body.^24,25^ These proteins are synthesized by hepatocytes in the liver and categorized as positive and negative acute-phase proteins in terms of changes in their concentrations. Serum albumin is considered as one of the negative acute-phase proteins produced by the liver and is considered an inflammatory marker.^7^ A large number of medical conditions such as inflammation, hepatic diseases and renal conditions result in decreases in serum albumin levels.^2,6^

Although the exact mechanism of the effect of periodontal disease on serum albumin levels is still unknown, it appears such relationship might be attributed to two hypotheses: the nutritional effects and the inflammatory effects of periodontal disease.^27^ Based on a study by Ogawa et al^7^ in 2006, the serum levels of albumin are associated with lower levels of total proteins, calcium, C-reactive protein and total cholesterol. Several studies have shown a relationship between individuals’ nutritional status ad serum albumin levels.^28,29^

A significant relationship has been reported between serum albumin levels and IgG concentration.^30^ Although C-reactive protein is not considered a nutritional index, it indicates the presence of inflammation in subjects with low albumin levels (31). Therefore, a decrease in serum albumin levels due to various infectious processes is associated with increases in the levels of C-reactive protein and IgG.^32,33^ In addition, serum albumin might serve as an antioxidative agent in the inhibition of free radicals.^34^ Low albumin levels might result in changes in early cellular damage and initiation or exacerbation of irreversible degenerative processes, which is considered a prerequisite for inflammatory, ischemic and proliferative conditions.^35^ All the above might explain the relationship between serum albumin levels and chronic periodontitis.

The results of the present study showed an inverse relationship between serum albumin levels and periodontal diseases. A study by Mojon et al^11^ in 1999 showed that subjects with a mean age of 85 years, who had pockets measuring >6 mm had significantly low serum albumin levels. Iwasaki et al^8^ carried out a study on 600 subjects, 70 years of age, to evaluate their periodontal status at baseline and after a year by considering attachment loss of ≥3 mm in one year as an indication of disease progression and concluded that serum albumin level is a good predictor for the evaluation of periodontal status in nonsmoking patients.

In a study by Ogawa et al^7^ the serum albumin levels were evaluated in 368 subjects with a mean age of 75 years and it was reported that an attachment loss of >6 mm significantly affected serum albumin levels.

In a study by Rajashri et al on 100 patients, 40‒70 years of age, in two healthy and periodontal disease groups with attachment loss of more than 5 mm, there was an inverse relationship between serum albumin levels and chronic periodontitis. The mean serum albumin levels in the healthy subjects and in the periodontitis group were 4.47±0.276 and 4.61±0.273 mg/dL, respectively.

The results of the present study confirmed the results of all the previous studies that have evaluated the relationship between serum albumin levels and periodontal disease. Only a limited number of similar studies are available. Anne et al^36^ and Radafshar et al^37^ reported that periodontal treatment induced a decrease in the serum levels of CRP. Graziani et al^38^ reported that serum fibrinogen levels returned to normal subsequent to non-surgical periodontal treatment in subjects with generalized chronic periodontitis. Chakraborty et al^17^ showed that non-surgical periodontal treatment in subjects with generalized chronic periodontitis gave rise to a decrease in ferritin serum levels. However, no study is available on the effect of non-surgical periodontal treatment on serum levels of negative acute-phase proteins.

A study by Shirmohammadi et al^39^ on the effect of non-surgical periodontal treatment on serum transferrin levels showed that 3 months after treatment, all the clinical periodontal parameters improved in association with an in increase in serum transferrin levels in the chronic periodontitis group. The present study is the first interventional study on the effect of non-surgical periodontal treatment on serum albumin levels. It seems logical that if periodontal inflammation gives rise to a decrease in serum levels of albumin subsequent to non-surgical periodontal treatment, resulting in a decrease in systemic inflammatory load, the serum albumin levels will increase, which might be explained by a decrease in the concentration of proinflammatory cytokines after periodontal treatment in subjects with chronic periodontitis.

The present study was a preliminary study and in order to achieve more definitive results, it is necessary to evaluate the relationship between serum levels of albumin and the concentrations of inflammatory cytokines such as TNF-α, IL-6 and IL-1 so that the relationship between inflammation and serum albumin levels will be further elucidated.

Further studies with larger samples sizes are necessary to determine whether serum levels of albumin can be used as a diagnostic factor for periodontal diseases.

## Conclusion

This study showed an inverse relationship between serum levels of albumin and chronic periodontitis; i.e., lower serum levels were reported in subjects with chronic periodontitis. Serum albumin levels increased after non-surgical periodontal treatment.

## Authors’ contributions

The study was planned by ASh and AS. Data collection was carried out by ZM; statistical analyses and interpretation of data were carried out by MS. The manuscript was prepared by AR and MF, and edited by AS. All the authors have read and approved the final manuscript for submission.

## Competing interests

The authors declare that they have no competing interests with regards to authorship and/or publications of this paper.

## Ethics approval

The study protocol was approved by the Ethics Committee in Medical Research of Tabriz University of Medical Sciences
